# From Photoluminescence Optimization to Green LED Fabrication: The Role of Molar Precursor Ratio in Carbon Dots

**DOI:** 10.3390/ma19040687

**Published:** 2026-02-11

**Authors:** Danilo Trapani, Filippo Saiano, Simona Boninelli, Sajeel Khan, Isodiana Crupi, Roberto Macaluso, Mauro Mosca

**Affiliations:** 1 Thin Films Laboratory, Department of Engineering, University of Palermo, IT 90128 Palermo, Italy; danilotrapani@tutanota.com (D.T.); sajeel.khan@polito.it (S.K.); isodiana.crupi@unipa.it (I.C.); roberto.macaluso@unipa.it (R.M.); 2 Department of Agricultural, Food and Forestry Sciences, University of Palermo, IT 90128 Palermo, Italy; filippo.saiano@unipa.it; 3 CNR-IMM, Department of Physics and Astronomy, University of Catania, IT 95123 Catania, Italy; simona.boninelli@ct.infn.it; 4 Department of Applied Science and Technology, Politecnico di Torino, IT 10129 Turin, Italy

**Keywords:** carbon dots, citric acid, urea, color conversion, green LEDs, photoluminescence quenching, solid-state luminescence

## Abstract

**Highlights:**

**What are the main findings?**

- Molar precursor ratio critically governs carbon dot photoluminescence efficiency.
- Optimized ratios suppress solid-state quenching in green-emitting carbon dots.
- Green carbon dots are successfully integrated as color-conversion layers in Light-Emitting Diodes (LEDs).

**What are the implications of the main findings?**

- Precursor ratio engineering enables reproducible, high-efficiency green emitters.
- Optimized carbon dots allow efficient solid-state color-conversion LED fabrication.
- The study links carbon dot synthesis directly to green LED device realization.

**Abstract:**

Carbon dots have emerged as promising luminescent materials for solid-state lighting and color-conversion applications; however, their photoluminescence efficiency in the solid state is often limited by aggregation-induced quenching phenomena. In this work, we systematically investigate the role of the molar precursor ratio on the optical properties of green-emitting carbon dots, with the aim of establishing a direct link between synthesis parameters, photoluminescence optimization, and device-level performance. By carefully tuning the precursor ratio during synthesis, a significant enhancement of photoluminescence intensity and a strong suppression of solid-state quenching are achieved while preserving spectral stability in the green region. The optimized carbon dots exhibit improved radiative recombination and favorable optical characteristics for solid-state integration. Building on these results, the carbon dots are successfully employed as color-conversion layers in the fabrication of green light-emitting diodes, demonstrating efficient green emission under electrical excitation. This study highlights precursor ratio engineering as a simple and effective strategy to tailor carbon dot photoluminescence and provides a clear pathway from materials optimization to the realization of green color-conversion LED devices.

## 1. Introduction

LEDs that convert colors have become widely used in various applications such as indoor and outdoor lighting [1], RGB displays [2], warning lights [3], automotive lighting [4], plant-growth lighting [5], and telecommunication [6]. These LEDs are preferred over other light sources due to their high efficiency, long lifetimes, low power consumption, high reliability, simple fabrication, and low cost [7].

Typically, the conversion layers in these LEDs consist of phosphors made from materials like cerium, erbium, yttrium, ytterbium, europium, and other rare earths [8,9,10,11]. Despite their name, rare earths are not actually rare and are commonly found together in numerous minerals [12] due to their similar ionic radii and predominant trivalent valency, which result in very similar chemical behavior. Extracting these materials often requires expensive techniques, yields low quantities, or involves the use of hazardous chemicals [13].

Recently, innovative nanostructured materials such as quantum dots (QDs) have been adopted for color conversion. These materials utilize the properties of quantum confinement to achieve highly efficient color conversion and can produce different conversion wavelengths from the same material [14]. Various fabrication methods, including self-assembly techniques [15], have been developed to reduce production costs. Moreover, QDs possess exceptional properties such as a broad absorption spectrum and tunable, spectrally narrow and highly efficient emission, allowing for customized emission spectra tailored to specific applications [16].

Unfortunately, QDs are typically composed of binary compounds such as lead sulfide [17], lead selenide [18], cadmium selenide [19], cadmium sulfide, and cadmium telluride [20], as well as indium arsenide [21], which are heavy metals and toxic materials. These substances pose a significant health risk to living beings due to their cytotoxicity [22].

However, carbon-core QDs, or simply carbon dots (CDs), offer a promising solution to these issues. Carbon is one of the most abundant and biocompatible elements, so CDs exhibit low toxicity, can be easily disposed of, and possess good water solubility and chemical stability [23]. Despite being carbon-based, CDs maintain excellent optical properties similar to inorganic QDs, including strong photoluminescence, high luminous stability, optical tunability, and a high-photoluminescence quantum yield [24,25,26].

Furthermore, CDs can be fabricated using various methods [27] and a wide range of precursors. These precursors can range from chemicals like urea (U) [28], citric acid (CA) [29], sodium citrate [30], glucosamine [31], ascorbic acid [32], ethanol [33], to everyday materials such as spent coffee grounds [34], milk, human hairs, onions, eggs, lemon, and more [27]. Typically, two precursors are utilized: a carbon source that contributes to the formation of the dot’s core and a source of functionalizing agents that bind to the surface of the dots, determining their optical properties.

Among the commonly used precursor combinations in the literature, the pair U:CA is widely employed due to its low cost, high availability, high yield in CD production, and the favorable optical properties of the resulting CDs [35,36,37,38,39].

Despite the extensive literature available on the fabrication of CDs [40], optical devices based on CDs are not so widespread due to the time-consuming [41] and costly processes required to make CDs suitable for solid-state applications. One of the major challenges is the filtration of CD solutions after carbonization, which is typically performed through dialysis [42,43,44]. This filtration method involves long waiting times, often lasting for days, and incurs high costs for membranes. Additionally, the use of specific matrices or unconventional approaches is necessary to address photoluminescence quenching issues commonly observed in solid-state organic materials [45]. These factors contribute to the complexity and expense of the overall process [46,47,48].

This study presents a simple, rapid, and cost-effective method for producing green LEDs with a CD-based color conversion layer using only U and CA. This approach eliminates the need for dialysis and any additional treatments to reduce quenching. It is worth noting that at a high U content, the carbonization process leads to the formation of cyanuric-acid-related or triazine-based species [49,50,51]. Therefore, these samples contain a heterogeneous system consisting of functionalized CDs and U-derived triazine-based species in addition to other molecules of unreacted precursors or carbonization sub-products. By optimizing the molar ratio of the precursors, we achieved the highest photoluminescence intensity in the liquid-state form. Subsequently, we fine-tuned the carbonization time to obtain solid-state luminescent CDs directly from the precursors. This methodology is particularly attractive to face the problem of the “green gap” in LEDs, that is, the lack of efficient green LEDs. To date, green LEDs are made of hexagonal III-N materials, but they reach just one-third of the wall-plug efficiency expected by the US Department of Energy roadmap in 2035 [52]. In other words, the current methods to fabricate green LEDs are inefficient and expensive [53]. This study demonstrates the feasibility of green LEDs in a simple and inexpensive way by using a composite of unfiltered CDs and urea-derived triazine-based species as color converters.

## 2. Materials and Methods

Different types of CDs were produced by adjusting the molar ratios between precursors. CA was utilized as the carbon source, while U served as the nitrogen source. CA (C_6_H_8_O_7_, MW = 192.13 g/mol) was procured from Acros Organics, while U (CH_4_N_2_O, MW = 60.06 g/mol) was from Carlo Erba Reagents; both are anhydrous. For liquid samples, a well-established method, which involves microwave-assisted carbonization [35,37,54], was followed. This approach enables the rapid synthesis of CDs both in liquid form and as solid-state CD-based layers suitable for color-conversion applications in green LEDs. A 10 mL aqueous solution of U:CA was subjected to microwave irradiation at 800 W, resulting in simultaneous carbonization and complete water evaporation within 6 min. During the process, the initially transparent solution gradually turned yellowish and subsequently darkened to black, indicating the formation of carbonaceous products. The evaporation of water yielded a solid hygroscopic material.

To ensure complete drying, the obtained solid was annealed in a muffle furnace at 130 °C for 45 min. The dried material was then manually ground using a pestle and further processed in a horizontally rotating test tube containing two steel spheres at 240 rpm for 1 h. This mechanical treatment resulted in fine powders with uniform grain size, suitable for subsequent optical characterization and device fabrication.

Similarly to other organic materials, CDs exhibit solid-state photoluminescence quenching [45]; therefore, the powders were dispersed in distilled water. A mixture of 50 mg of CDs powder and 20 mL of water was vigorously stirred at 4000 rpm for 1 h, followed by a resting period of approximately 15 h to allow for the precipitation of larger impurities and quenched aggregates. Subsequently, the dispersion was filtered using a 0,45 µm Millipore syringe filter; then, different aqueous solutions of water-dispersed CDs at different precursor molar ratios were produced.

Unless otherwise specified, all samples discussed in this work were synthesized using a fixed microwave irradiation time of 6 min; shorter irradiation times (60 s, 90 s, and 120 s) were intentionally employed only for the additional experiments described in Section 3.4 to investigate the effect of reduced carbonization time at a fixed precursor molar ratio (U:CA = 100:1).

The dialysis step was intentionally omitted in this work to preserve U-derived molecular species that contribute to solid-state emission; this choice is specific to the present application-oriented study and should not be generalized to CD synthesis aimed at chemical or spectroscopic identification.

The transmittance measurements were conducted by a DMS-90 Varian spectrophotometer in the wavelength range from 400 nm to 700 nm.

Photoluminescence spectra were obtained by illuminating the cuvette with a 404 nm laser at an angle of 45° and employing an ARC SpectraPro-275 monochromator and a PD-438 photomultiplier tube. A Tektronix TDS 1012 digital oscilloscope was utilized for spectrum acquisition. The light was focused on the monochromator through an optical lens (7 cm diameter, 30 cm focal length). The photomultiplier tube, biased at 1200 V, collected the light coming from the output slit of the monochromator. The provided current signal was then displayed on the oscilloscope using a 1 kΩ resistor. To ensure minimal background noise, all the measurements were carried out in a dark environment.

The photoluminescence detection system was calibrated using a Newport optical power meter (Model 1815-C, Newport Corporation, Irvine, CA, USA) equipped with a calibrated UV-enhanced large-area photodiode. The calibration was performed under the same excitation conditions used for photoluminescence measurements, allowing the conversion of the PMT signal into absolute optical power. In addition, the spectral response of the setup was verified using interference band-pass filters centered at 454 nm and 540 nm (Thorlabs), corresponding to the main emission wavelengths of the investigated samples.

Photoluminescence intensities are reported for comparative purposes only, as all measurements were performed under identical excitation and detection conditions; absolute quantum yield measurements were not performed.

Time-resolved photoluminescence measurements were performed using a FLIM Starter Kit (FLIM Labs) based on time-correlated single-photon counting (TCSPC). The samples were excited using a picosecond pulsed laser source, and the emitted photoluminescence was collected and detected by a single-photon avalanche diode. The instrument response function was measured separately and taken into account during data analysis. Photoluminescence decay curves were fitted using an exponential decay model, allowing the extraction of the characteristic photoluminescence lifetime.

## 3. Results and Discussion

Polymethyl methacrylate cuvettes with a 1 cm^2^ base area were filled with different filtered water dispersions of CDs, and the transmittance spectra of all the samples were acquired. The photoluminescence intensities of the samples were measured and compared to each other. Since all the samples exhibit high absorption in the near-UV–violet region, a 404 nm laser diode was used as a pump source. Although UV-vis absorption spectroscopy can provide additional insight into optical transitions, in this work, absorption at the excitation wavelength (404 nm) is directly addressed through transmittance measurements, which are the most relevant for the analysis of photoluminescence under the employed excitation conditions. Furthermore, infrared and structural characterizations were carried out to identify the parameters for an optimal color conversion.

### 3.1. Precursor Molar Ratios Investigation

**Figure 1:**
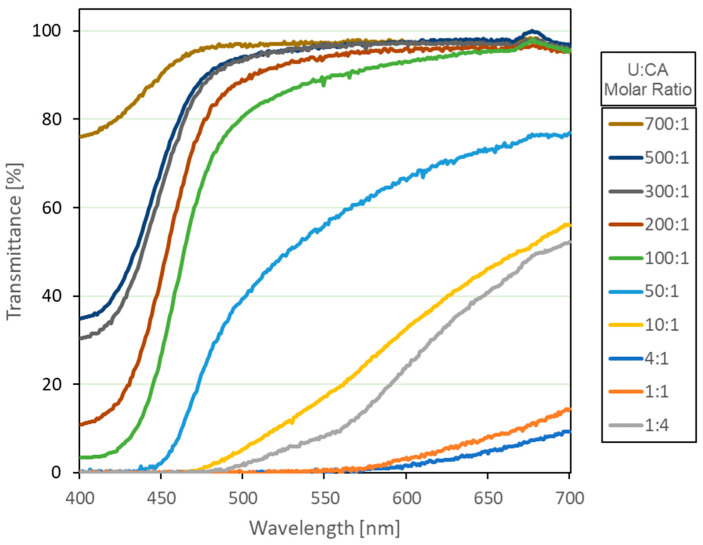
Transmittance spectra of CD aqueous solutions, with U:CA molar ratios spanning from 1:4 to 700:1.

Figure 1 shows the transmittance spectra of different CD solutions at different U:CA molar ratios (1:4, 1:1, 4:1, 10:1, 50:1, 100:1, 200:1, 300:1, 500:1, 700:1). All the samples present a cutoff wavelength as short as approximately 450–470 nm. These samples are quasi-transparent for wavelengths longer than the cutoff wavelength. One should note that the cutoff wavelength shifts towards shorter wavelengths with the precursors’ ratio. The samples with low U:CA molar ratios exhibit low transmittance values in a wide wavelength range, while a significant increase in the transmittance is observed for U:CA ratios larger than 50:1. Below this value, the solutions appeared brownish and opaque, while over it, the samples are quite transparent and yellowish. Indeed, when the ratio between U and CA is below one, with CA exceeding the U, the transmittance slightly increases again.

It should be noted that a near-zero transmittance at 404 nm indicates strong absorption by non-radiative species rather than efficient light conversion, which explains the low photoluminescence observed at low U:CA ratios.

**Figure 2:**
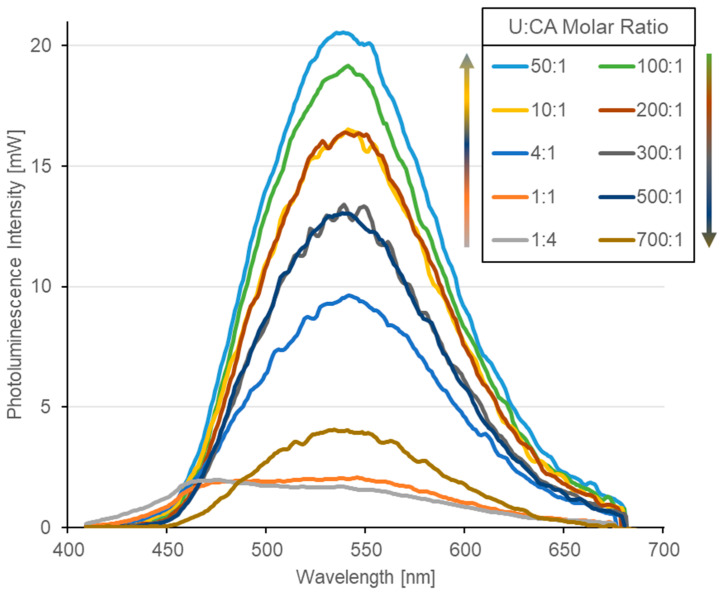
Photoluminescence spectra of CDs in aqueous solutions for different U:CA molar ratios ranging from 1:4 to 700:1. The CDs were optically excited using a 404 nm laser.

To identify the optimal photoluminescence properties for the fabrication of CD-based LEDs using U and CA as precursors, an initial molar ratio of 1:1 was investigated. Subsequently, the precursor composition was systematically varied by introducing an excess of U (up to 700:1) or CA (up to 1:4) in order to determine the most favorable synthesis conditions. As shown in Figure 2, U-rich samples exhibit a markedly higher photoluminescence intensity, with a dominant emission peak centered at 540 nm. In contrast, samples prepared with 1:4 and 1:1 molar ratios display comparable photoluminescence intensities but show a pronounced blue shift in the emission peak to approximately 464 nm. This spectral shift is also evident to the naked eye, with these samples exhibiting a bluish emission compared to the green emission observed in U-rich samples. A direct comparison of the photoluminescence spectra (Figure 2) further reveals that the sample synthesized with a 50:1 molar ratio provides the highest photoluminescence intensity among the investigated compositions.

**Figure 3:**
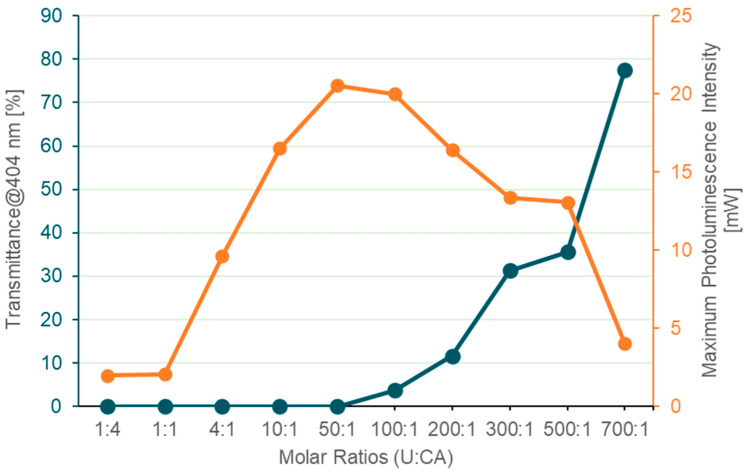
Comparison between the transmittance at 404 nm and the maximum photoluminescence intensity at 540 nm as a function of the U:CA molar ratio.

Figure 3 shows the photoluminescence intensity peaks, expressed in mW, as a function of the precursors’ molar ratio. Additionally, the behavior of the transmittance at a pump wavelength of 404 nm is also reported by relating the amount of light transmitted and converted. Additionally, the transmittance at the pump wavelength (404 nm) is reported and compared with the corresponding photoluminescence intensity in order to qualitatively correlate the fraction of excitation light absorbed by the sample with the emitted photoluminescence. Figure 3 clearly demonstrates that below a ratio of 50:1, the pump radiation is completely absorbed, and the photoluminescence initially increases with the U content; when the ratio exceeds 50:1, the U excess leads to a decrease in photoluminescence intensity. However, given that the transmittance increases with the U content, the mechanism of CD formation is supposed to be less efficient.

In more detail, the CDs have a complex band-gap structure, not only originating from the presence of cyanuric-acid-related or triazine-based species, but also functional groups and defect-related traps with emissive properties. The huge number of C=N and C-N bonds generated by the carbonization process, combined with the products of the thermal reaction, results in a complex band-gap structure distribution. On one hand, U allows the creation of N-functionalized CDs, but on the other hand, especially at high U content, which promotes the formation of urea-derived triazine-based species. In other words, the product of carbonization is mainly composed of N-doped CDs and U-derived triazine-based molecular species. Also, it is worth considering that the thermal U:CA reaction is quite complex, and lots of reaction products are created. It is proven that CDs with the core carbon nanoparticles functionalized by HPPT (4-hydroxy-1H pyrrolo [3,4-c] pyridine-1,3,6 (2H,5H) -trione) are normally produced during the U:CA reaction, and the HPPT functionalization is considered as being mainly responsible for the CD luminescence [55,56]. However, even the formation of exciplex-like states between CDs (as donors) and cyanuric-acid-related or triazine-based species (as acceptors) leads to a fluorescence phenomenon due to the radiative recombination of the exciplex [57].

**Figure 4:**
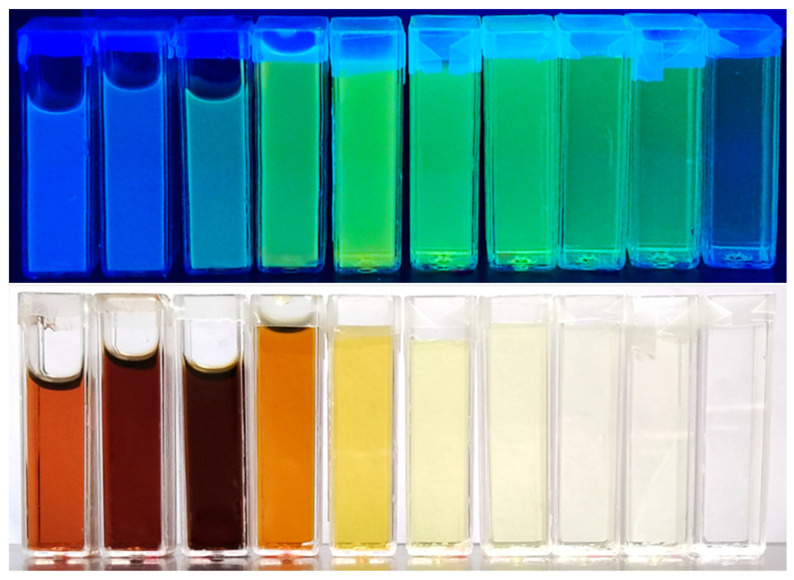
Photographs of aqueous CD dispersions with different U:CA molar ratios (1:4, 1:1, 4:1, 10:1, 50:1, 100:1, 200:1, 300:1, 500:1, and 700:1, from left to right) under UV illumination (**top**) and ambient light (**bottom**).

These optical characteristics are clearly illustrated in the photographs shown in Figure 4. Under ambient light illumination, the color of the aqueous CD dispersions ranges from dark opaque brown for the 1:4, 1:1, and 4:1 samples to pale, transparent yellow for U-rich compositions (100:1 and above). When exposed to UV excitation, the CA-rich samples (1:4 and 1:1) exhibit blue luminescence, which progressively shifts toward green emission as the U content increases, reaching the highest intensity for the 50:1 sample.

Luminescence properties are strongly dependent on the features of the CD–matrix composite, where the matrix is formed by urea-derived triazine-based species, and, consequently, on the U:CA molar ratio. When U is brought to high temperatures, it can produce cyanuric acid [58], so a high content of U in the solution can turn into an excess of cyanuric acid with respect to CDs. When CDs interact with a surrounding matrix formed by cyanuric-acid-related or triazine-based species, the radiative emissions come not only from the CDs themselves but also from radiative transitions between states of the matrix and of the CDs. In some cases, transitions can occur between singlet states of one and triplet states of the other, which explains the observed phosphorescence in some other CDs present in the literature [59]. Moreover, in U-rich samples, the emission can originate both from interactions between the CDs and the surrounding matrix and from the matrix itself. In the present samples, however, the observed emission is purely fluorescent, with nanosecond-scale lifetimes and no detectable delayed component. The resulting emission is predominantly located in the green spectral region. Time-resolved measurements indicate a rapid decay of the emission, with lifetimes on the order of tens of nanoseconds, confirming that the observed luminescence arises from fluorescence rather than phosphorescence, as no delayed emission is detected after the excitation is switched off.

**Figure 5:**
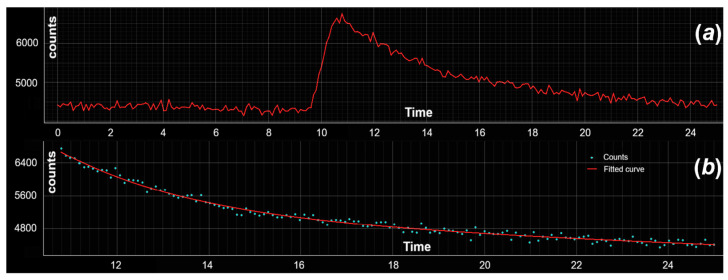
(**a**) Time-resolved photoluminescence response of the carbon-dot-based sample under picosecond pulsed laser excitation. (**b**) Photoluminescence decay curve fitted using a single-exponential model $A\cdot e^{-t/\tau }+B$, yielding a lifetime τ = 3.88 ns. The nanosecond-scale lifetime confirms the fluorescent nature of the emission and excludes phosphorescence contributions.

Time-resolved photoluminescence measurements were performed to further elucidate the nature of the emissive processes and to verify the absence of phosphorescence contributions. Figure 5 reports the temporal response of the sample following excitation with a picosecond laser pulse, while Figure 5 shows the corresponding photoluminescence decay curve.

The decay dynamics are well described by a single-exponential model of the form $A\cdot e^{-t/\tau }+B$, where τ represents the characteristic photoluminescence lifetime. The fitting yields a lifetime τ = 3.88 ns, which is fully consistent with fluorescence originating from molecular or surface-related emissive states and from matrix-assisted radiative recombination involving N-doped carbon dots.

The absence of any long-lived decay component or delayed emission clearly rules out phosphorescence or triplet-mediated emission processes. This result supports the interpretation that the observed green emission arises from prompt radiative recombination pathways, in agreement with the proposed emission mechanisms and with the steady-state photoluminescence behavior discussed above.

In this context, cyanuric acid plays a key role in enhancing the green photoluminescence emission [57]. This could explain the shift in the peak emission wavelength. In contrast, when there is an excess of CA or a low U:CA ratio, cyanuric acid is produced in negligible amounts or not at all. Without the matrix, the emission occurs only between CD states, resulting in a blue emission. The cyanuric acid matrix also helps prevent aggregation-caused quenching, and this is clearly visible in our series of samples. However, if the U concentration exceeds a 50:1 ratio, the balance between the matrix and the CDs is messed up, leading to a decrease in the CD population and a subsequent drop in emission intensity. Furthermore, an excess of cyanuric acid is reported to have a detrimental effect in terms of the number of photons emitted by the CDs [60]. Photoluminescence quenching in these cases is attributed to the inhibition of the conjugated π electrons [60]. At a higher molar ratio, the quenching effect due to the presence of excessive N and O functional groups causes the photoluminescence intensity to be further reduced. The reason is that the cyanuric acid partially blocks the transfer of π-conjugated electrons, and the high density of N and O dopants makes the energy transfer ineffective due to many zigzag paths between the dopant and the CD surface [58]. Moreover, the excess of U increases the pH of the solution, which causes the breaking of the amide bonds in the HPPT fluorophores; in fact, an alkaline environment fosters the hydrolyzation of the amide bonds and quenches the fluorescence [56].

Although different emissive pathways are discussed, they should not be regarded as mutually exclusive. Instead, the photoluminescence behavior of the U-CA system reflects the evolution and relative contribution of molecular fluorophores, surface states, and matrix-assisted emission as a function of the precursor ratio and carbonization conditions [61]. Under optimized synthesis conditions, the dominant contribution to green emission is attributed to matrix-assisted radiative recombination involving N-doped CDs interacting with U-derived triazine-based species, while HPPT-related emission is expected to play a secondary role.

Overall, the formation of emissive CDs in the U-CA system should be regarded as an evolving process jointly controlled by the precursor molar ratio and carbonization time. Low U content or short carbonization times favor molecular and surface-related emissive states, typically associated with blue emissions. Increasing the U content promotes nitrogen incorporation and the formation of U-derived triazine-based species, which interact with N-doped CDs and progressively shift the emission toward the green region. Carbonization time further regulates the balance between molecular fluorophores and carbonaceous domains: insufficient carbonization results in weak emission, whereas excessive carbonization suppresses photoluminescence due to over-carbonization. An intermediate carbonization regime yields the optimal balance between CD formation and matrix-assisted radiative recombination, explaining the observed evolution of photoluminescence intensity and spectral position.

### 3.2. Infrared Analysis

The Attenuated Total Reflection-Fourier Transform Infrared Spectroscopy (ATR-FTIR) technique was employed to investigate the behavior of the mixtures obtained with different U:CA ratios in relation to the photoluminescence phenomenon. The surface functional groups were characterized using a spectrophotometer Spectrum Two equipped with KBr optics and an ATR diamond cell from PerkinElmer Inc., Wellesley, MA, USA. The analysis was conducted on powder samples at room temperature, with a scanning range of 4000 to 400 cm^−1^ and a resolution of 2 cm^−1^. Each sample underwent 32 scans, and the resulting spectra were processed using the Spectrum 10 software from PerkinElmer Inc., Wellesley, MA, USA.

**Figure 6:**
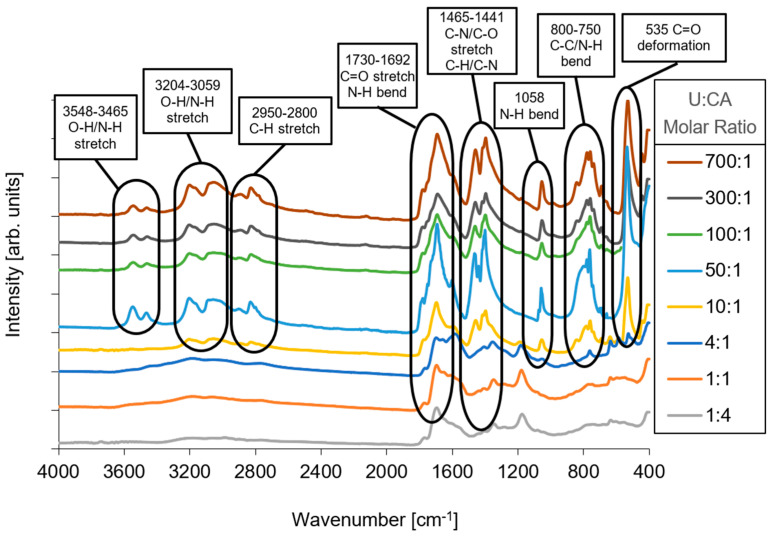
FTIR spectra of CDs synthesized with U:CA molar ratios ranging from 1:4 to 700:1, with the assignment of the main absorption bands to the corresponding chemical bonds.

The more characteristic features of the samples are shown in Figure 6. The bands observed at 3548, 3465, 3204, and 3059 cm^−1^ indicate the stretching of O-H and N-H bonds, also interacting through hydrogen bonding. The bands within the range of 2950–2800 cm^−1^ can be attributed to C-H stretching. At 1730 and 1692 cm^−1^, two prominent bands emerge, indicating the stretching of C=O carboxylic groups and the bending of N-H bonds (1692 cm^−1^). Additionally, at 1602 cm^−1^ there is an increase in the band associated with the stretching of amidic C=O or C=N bonds. Other notable features include the peak at 1465 cm^−1^, which corresponds to C-N stretching, and the peaks at 1441 and 1401 cm^−1^, which are attributed to the stretching of C-O bonds and the bending of C-H and C-N bonds. At lower frequencies, peaks at 1058, 800, 762, and 535 cm^−1^ are visible. The peak at 1050 cm^−1^ is probably due to N-H bending, while the peaks within the range of 800–750 cm^−1^ can be attributed to C-C and N-H bending. Lastly, the peak at 535 cm^−1^ is likely a result of a C=O out-of-plane deformation. References [62,63] support the features and characteristics described in this section.

The ATR-FTIR spectra of different samples exhibit varying behavior depending on the molar ratios employed. Samples with U:CA molar ratios of 1:4 and 1:1 display similar spectra. In contrast, those with ratios of 10:1 and 50:1 seem to indicate the formation (10:1) and stabilization (50:1) of triazine-based, cyanuric-acid-related species, yet suggested by the transmittance and photoluminescence data, as also supported by similar studies [49,50,51,58,64]. It is evident that an increase in the U:CA ratio, up to 700:1, after washing the sample, does not exhibit, in the ATR-FTIR spectra, any discernible differences in the profile or ratio between the peaks. Also, in not-washed samples, the excess of U leads to the formation of nitrogen-rich, triazine-based species, which are known to play a role in the quenching behavior of CDs. This indicates that the increase in U amounts does not impact the chemical properties of the compound obtained at a 50:1 ratio. Also, Carbon-13 Nuclear Magnetic Resonance (^13^C-NMR) measurements in solution (data reported in Supplementary Materials) support the ATR-FTIR results.

Finally, an important feature that can be accomplished by the spectra in Figure 6 is that several peaks, which are only detected for U:CA ratio values greater than 10:1 (such as 1058 cm^−1^, 1441 cm^−1^, 1465 cm^−1^), are also characteristic of triazine-based structures, including cyanuric-acid-related species.

### 3.3. Structural Analysis

An electron microscopy investigation was conducted using a ZEISS Supra 25 Scanning Electron Microscope (SEM) (Zeiss, Oberkochen, Germany) and a JEOL JEM 2010F Transmission Electron Microscope (TEM) (JEOL, Akishima, Tokyo, Japan). To minimize sample degradation, the electron beam voltage was reduced to 3 keV for the SEM analysis. For the TEM analysis, the instrument operated at 200 keV. Prior to the analysis, a few drops of the liquid solution of CDs with molar ratios of 50:1 were applied onto a TEM 300 mesh grid that was coated with a lacey carbon film. The samples were then dried at room temperature.

**Figure 7:**
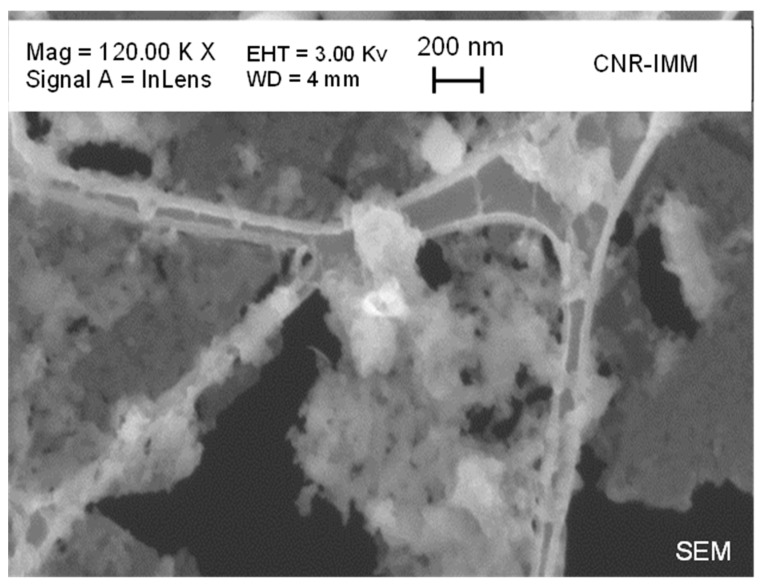
SEM image showing a cluster of CDs distributed within the solid-state matrix.

**Figure 8:**
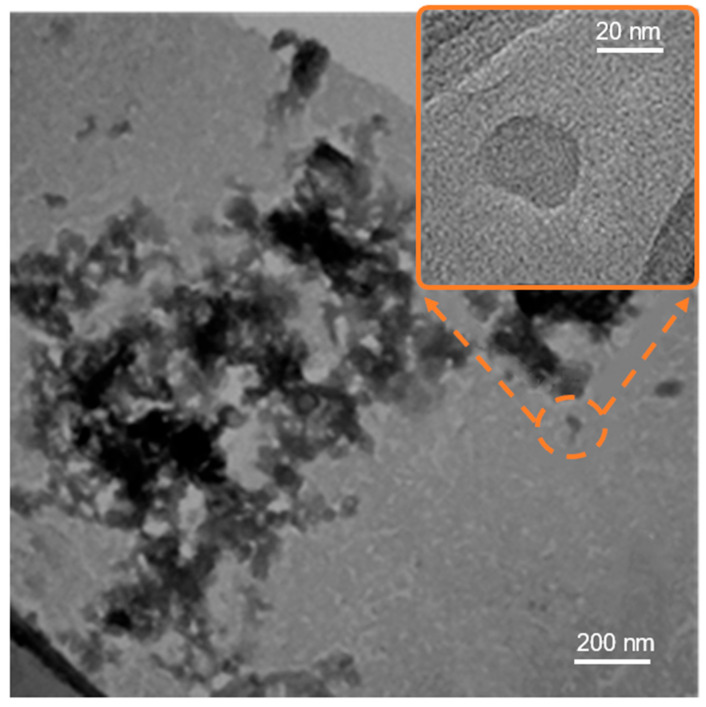
BF-TEM image of the sample synthesized with a U:CA molar ratio of 50:1, showing amorphous CDs forming aggregates. The inset shows a high-magnification image of a single amorphous CD.

**Figure 9:**
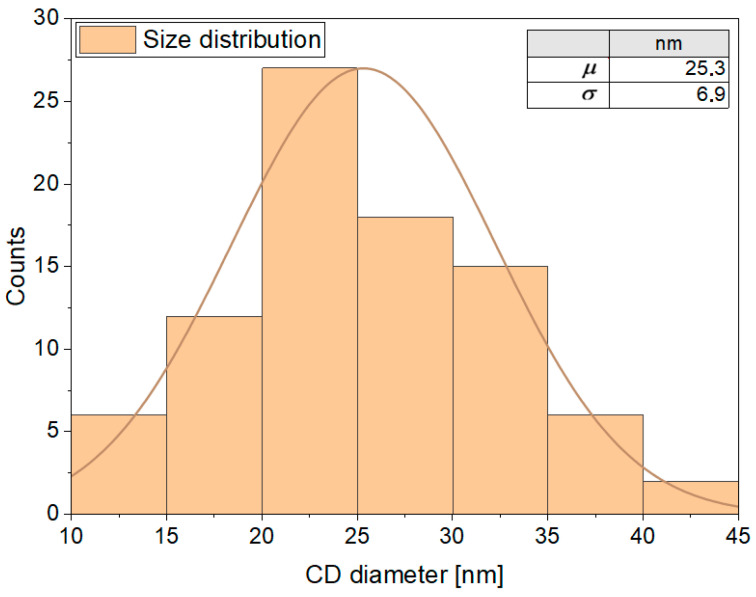
Size distribution histogram obtained from TEM images, together with a Gaussian fit. The extracted average diameter is *μ* = 25.3 nm with a standard deviation *σ* = 6.9 nm (values reported in the table in the upper right corner).

SEM analysis (Figure 7) does not aim at determining the size of individual CDs but rather provides information on the mesoscopic morphology of the material. The image reveals that the CDs are not present as isolated particles but form interconnected aggregates and network-like structures distributed on a quasi-continuous solid-state layer. This aggregation behavior is consistent with the absence of a dialysis step and with the coexistence of CDs and U-derived molecular species, which promote clustering and solid-state network formation. Bright-field TEM analysis (BF-TEM) (Figure 8) confirms the presence of amorphous CDs forming aggregates. In order to provide a statistically meaningful size evaluation, a size distribution analysis was performed by measuring the diameter of a representative number of CDs extracted from TEM images (Figure 9). As shown in Figure 8, the BF-TEM image displays an aggregate formed by multiple CDs. The inset provides a high-resolution image of a single amorphous CD with a mean size of 25.5 nm, which is representative of the amorphous phase observed across the CD population. The amorphous nature of the CDs is further confirmed by electron diffraction analysis.

BF-TEM analysis reveals the presence of relatively large CDs, with sizes exceeding 20 nm, which is consistent with the absence of a post-synthesis dialysis step. As a result, smaller-sized CDs are present in lower concentrations and are more difficult to detect. The relatively large average size of the CDs indicates that the observed photoluminescence is unlikely to be dominated by quantum confinement effects. Instead, the emission is more plausibly associated with the formation of different chemical bonds and molecular states involving the CDs and surrounding species, such as urea-derived triazine-based compounds.

This interpretation is further supported by FTIR measurements, which show a marked shift in the position of the main absorption bands as the U:CA molar ratio increases from 4:1 to 10:1 and higher. Since the location of these bands is directly related to specific molecular bonds, the observed shifts suggest the formation of distinct bonding configurations and possibly different molecular species, each characterized by different electronic transition levels.

Additional evidence for this mechanism is provided by the photoluminescence behavior shown in Figure 4, where a clear spectral transition is observed from blue emission in CA-rich samples (1:4 and 1:1) to a dominant green emission in U-rich compositions (4:1–700:1).

The resulting size distribution in Figure 9 shows a dominant population of CDs with diameters ranging from approximately 18 to 32 nm, corresponding to *μ* ± *σ*, where *μ* is the average diameter and *σ* the standard deviation. Smaller CDs may still be present; however, their contribution is minor, as indicated by the Gaussian distribution, and they are not easily detectable by TEM due to their low relative abundance and contrast. The prevalence of relatively large, amorphous CDs is consistent with the synthesis route adopted in this work, particularly with the omission of dialysis, which is known to selectively remove larger aggregates and reaction by-products.

It is worth noting that CDs with sizes larger than 10 nm have been widely reported in the literature, especially for amorphous or molecular-state-dominated CDs, where photoluminescence is not governed by quantum confinement effects but rather by surface states, functional groups, and molecular fluorophores.

### 3.4. Preparation of CDs for Color-Conversion Green LEDs

Although the sample synthesized with a U:CA molar ratio of 50:1 exhibits the highest photoluminescence intensity, the 100:1 sample offers a more favorable trade-off between photoluminescence efficiency and optical transmittance. For this reason, CDs prepared with a 100:1 molar ratio were selected for further investigation as potential color-conversion materials for phosphor-based green LEDs. To evaluate the influence of the carbonization process on the optical properties, three additional samples were synthesized by varying the microwave irradiation time to 60 s, 90 s, and 120 s while keeping the precursor molar ratio fixed at 100:1.

During microwave irradiation, the aqueous U:CA solution exhibited a progressive change in color, suggesting the gradual evolution of the carbonization process. Accordingly, the optical properties of the samples were systematically analyzed at different stages of synthesis, including before, during, and after microwave treatment. Immediately after irradiation, all samples were in liquid form; however, upon cooling, the viscosity increased and the materials progressively solidified. In addition, a clear variation in sample color was observed as a function of irradiation time, evolving from a whitish appearance to a progressively darker brown, indicating increasing carbonization.

**Figure 10:**
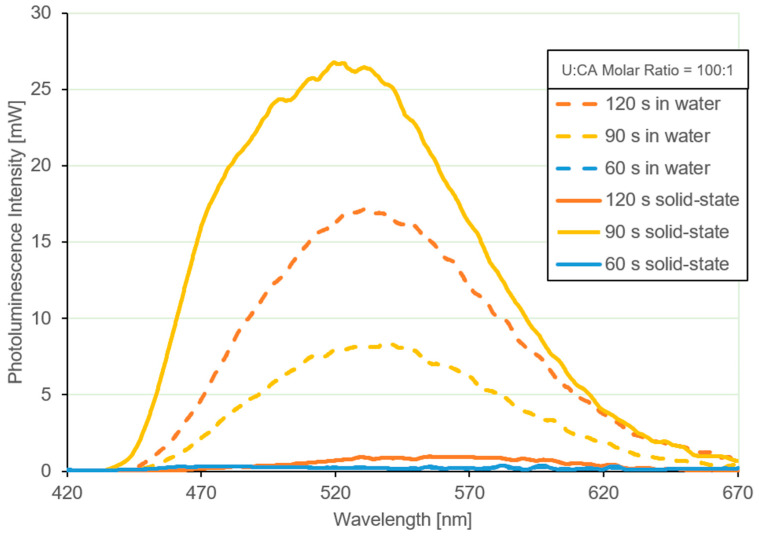
Photoluminescence spectra of solid-state CDs and corresponding aqueous dispersions prepared at different carbonization times (U:CA = 100:1), excited at 404 nm.

Interestingly, the samples subjected to longer microwave irradiation times exhibited solid-state photoluminescence when excited with the same 404 nm laser used in previous measurements. Figure 10 compares the photoluminescence behavior of the three samples both in the solid state and when dispersed in water. Among them, the sample irradiated for 90 s shows a markedly enhanced photoluminescence intensity in the solid state compared to both its liquid dispersion and the other solid samples. Notably, when this sample is dispersed in water, a significant reduction in photoluminescence intensity is observed.

In contrast, the sample irradiated for 60 s does not exhibit detectable luminescence, indicating insufficient carbonization. The sample irradiated for 120 s displays photoluminescence properties comparable to those of samples prepared using longer heating times (6 min), as discussed in the previous sections. In addition, a slight blue shift in the emission peak is observed for the 90 s sample relative to the 120 s sample, suggesting subtle differences in the emissive states induced by the carbonization time.

As reported in [56], the thermal reaction between CA and U leads to a progressive increase in carbon content with increasing reaction time, resulting in a higher density of CDs. However, prolonged carbonization, especially in the presence of a high U content, tends to suppress and quench photoluminescence, as the conditions required for the formation of HPPT are no longer fulfilled.

Conversely, a relatively short microwave treatment time (90 s) produces a lower concentration of CDs that are effectively dispersed within a solid matrix formed by cyanuric-acid-related or triazine-based species. This configuration limits aggregation-induced quenching mechanisms and results in enhanced luminescence, particularly in the solid-state form. Conversely, below a critical minimum heating time, the U:CA reaction does not generate sufficient carbon to form CDs, and the resulting samples exhibit little to no detectable photoluminescence.

The properties of the sample synthesized with a microwave irradiation time of 90 s make it a particularly suitable candidate for color-conversion applications in LED devices. An additional advantageous feature is that, upon heating the solidified material to approximately 120 °C, it reverts to the liquid state without any detectable alteration of its optical properties. This thermoreversible behavior enables straightforward processing using conventional deposition techniques such as spin-coating or dip-coating.

**Figure 11:**
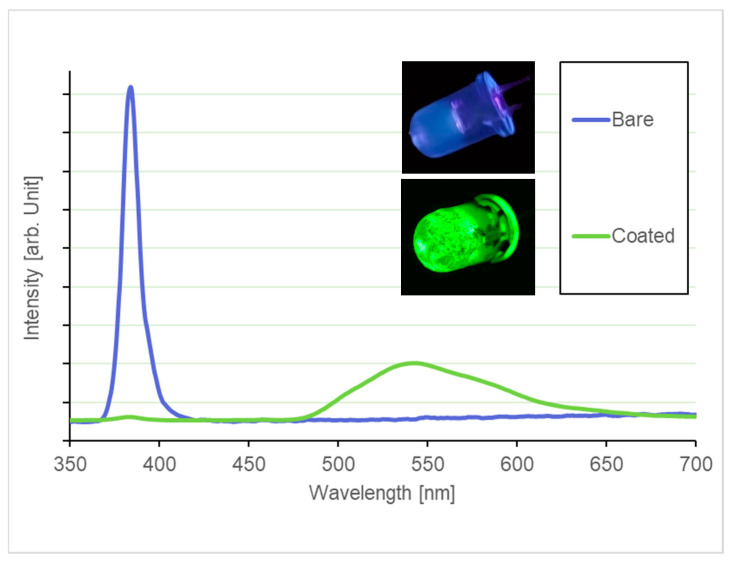
Emission spectra of a commercial 385 nm LED before and after dip-coating with the 90 s CD sample. The inset shows photographs of the LED prior to (top) and after (bottom) the color-conversion coating.

To validate this approach, a thin film of the 90 s CD sample was deposited onto a commercial 385 nm LED by dip-coating. As shown in Figure 11, the resulting emission spectrum exhibits a clear red shift, with a dominant peak centered at approximately 540 nm. The spectra were acquired using a calibrated spectrometer (Model HR4000CG-UV-NIR, Ocean Optics, Dunedin, FL, USA). The strong luminescence observed in the solid state indicates that the host matrix effectively separates the CDs, thereby suppressing aggregation-induced quenching effects.

For comparison, photoluminescence measurements were also performed on samples composed solely of CA or U. In both cases, no emission was detected under optical excitation, confirming that the observed luminescence originates exclusively from the CDs and the products of the U:CA thermal reaction.

This result is particularly relevant because it demonstrates that color-conversion green LEDs, such as the one shown in Figure 11, can be fabricated through a simple and rapid processing route, without the need for dialysis or complex post-synthesis treatments. The use of readily accessible processing steps enables a significant reduction in both fabrication time [41] and overall cost [65], highlighting the potential of the proposed approach for scalable and cost-effective green LED manufacturing.

## 4. Conclusions

In this work, we demonstrated that the limitations commonly affecting the solid-state photoluminescence of CDs can be effectively addressed through a rational optimization of the synthesis conditions. In particular, fine tuning of key fabrication parameters, such as the precursors’ molar ratio and the carbonization time, was shown to play a decisive role in governing the optical properties of green-emitting CDs. Even minor variations in these parameters result in significant changes in emission intensity and spectral features, highlighting the sensitivity of CD photoluminescence to synthesis boundary conditions.

The strong dependence of the emission behavior on the precursors’ molar ratio suggests that multiple radiative mechanisms contribute to the observed fluorescence, likely arising from interactions between CDs, surface functional groups, and U-derived triazine-based reaction by-products, rather than from size-related quantum confinement effects. Time-resolved photoluminescence measurements further support this interpretation by revealing a nanosecond-scale lifetime (τ = 3.88 ns), consistent with prompt fluorescence arising from chemical and matrix-assisted radiative pathways and confirming the absence of phosphorescence or long-lived emission processes. Furthermore, our results indicate that excessive carbonization of the precursors is not required to obtain efficient luminescent CDs, while the careful selection of an appropriate carbonization time is crucial for maximizing solid-state photoluminescence.

Importantly, the optimized synthesis conditions enable the achievement of efficient solid-state emission without the need for post-synthesis treatments such as dialysis or the incorporation of external matrices. As a result, the produced CDs are directly usable as color-conversion materials and were successfully employed in the fabrication of green LEDs. This work therefore provides a simple, scalable, and environmentally friendly route that directly connects CD synthesis optimization to the realization of green color-conversion LED devices.

## Data Availability

The original contributions presented in this study are included in the article/Supplementary Material. Further inquiries can be directed to the corresponding author.

## Supplementary Materials

The following supporting information can be downloaded at: https://www.mdpi.com/article/10.3390/ma19040687/s1, Figure S1: ^13^C NMR spectrum of CDs synthesized with a U:CA molar ratio of 50:1.

## Abbreviations

The following abbreviations are used in this manuscript:

QD	Quantum dot
CD	Carbon dot
U	Urea
CA	Citric acid
HPPT	4-hydroxy-1H pyrrolo [3,4-c] pyridine-1,3,6(2H,5H)-trione
ATR-FTIR	Attenuated Total Reflection-Fourier Transform Infrared Spectroscopy
^13^C-NMR	Carbon-13 Nuclear Magnetic Resonance
SEM	Scanning Electron Microscope
TEM	Transmission Electron Microscope
BF-TEM	Bright-Field Transmission Electron Microscope
