# Supplementary material for: From Photoluminescence Optimization to Green LED Fabrication: The Role of Molar Precursor Ratio in Carbon Dots
Source: Materials (Basel). 2026 Feb 11;19(4):687. doi: 10.3390/ma19040687 (PMC12942580; doi:10.3390/ma19040687)
Supplement: Supplementary file 1 [file materials-19-00687-s001.zip › materials-4100750-supplementary.pdf]

materials

Supplementary Materials

From Photoluminescence Optimization to Green LED Fabrication: The Role of Molar Precursor Ratio in Carbon Dots

Danilo Trapani[1], Filippo Saiano[2], Simona Boninelli[3], Isodiana Crupi[1], Roberto Macaluso[1], Mauro Mosca[1,*]

[1] Department of Engineering, University of Palermo (Thin Films Laboratory), IT 90128, Palermo, Italy
[2] Department of Agricultural, Food and Forestry Sciences, University of Palermo, IT 90128, Italy
[3] CNR-IMM, Department of Physics and Astronomy, University of Catania, IT 95123 Catania, Italy
* Correspondence: mauro.mosca@unipa.it

^{13}C-NMR characterization

^{13}C NMR analysis was performed on the CD sample synthesized with a U:CA molar ratio of 50:1. Spectra were acquired using a Varian 500 MHz magnet equipped with a ONEnmr probe with gradients and a ProPulse Varian console. Data acquisition and processing were carried out using vNMRj software running on a Linux CentOS workstation. Samples were prepared in 5 mm NMR tubes using 500–700 µL of solution at an approximate concentration of 50 mM. Due to their water solubility, CDs were analyzed in D_2O without further purification of the deuterated solvent.

NMR experiments were conducted using a calibrated 90° pulse, with 16 scans, an acquisition time of 2.5 s, and a relaxation delay of 2 s. $^{13}C\{^1H\}$ spectra were recorded at 125.73 MHz and 27 °C, with a spectral width of 200 ppm. Chemical shifts were referenced externally to tetramethylsilane (TMS, δ = 0.00 ppm).

The ^{13}C NMR spectrum shows signals at 35.10, 38.20, and 39.90 ppm, which are characteristic of sp^3-hybridized carbon atoms bonded to hydrogen or alkyl groups ($–CH_2$ and $–CH_3$). These features indicate the presence of aliphatic carbon domains within the CD structure. A resonance at 53.14 ppm can be attributed to carbon atoms bonded to electronegative atoms such as oxygen or nitrogen, suggesting methoxy ($–OCH_3$) or amine ($–CH_2–N–$) functionalities. Additional signals at 59.49 and 61.53 ppm are consistent with carbon atoms bonded to multiple electronegative atoms, as commonly observed in ether or primary alcohol environments ($–CH_2OH$ or $–CH_2O–$), supporting the presence of oxygen-containing surface functional groups that contribute to the hydrophilic character of the CDs.

Signals observed at higher chemical shifts, namely at 152.37 and 160.51 ppm, fall within the aromatic and conjugated carbon region and are typically associated with sp^2-hybridized carbon atoms bonded to heteroatoms such as nitrogen or oxygen. Furthermore, resonances at 165.19, 169.90, and 179.16 ppm are indicative of carbonyl carbon atoms (C=O), commonly found in amides, esters, or carboxylic acid groups. These features point to a significant degree of surface oxidation and functionalization, which is consistent with the observed solubility and optical behavior of the CDs.

Overall, the NMR data suggest that the CDs consist of a mixed sp^2/sp^3 carbon framework with a high density of oxygen- and nitrogen-containing functional groups, including alcohols, ethers, amines, and carbonyl species. Such surface functionalization is typical of CDs synthesized from citric acid and urea and plays a key role in governing their optical properties.

Regarding the possible presence of HPPT-related structures, the combination of aromatic/conjugated carbon signals (above 150 ppm) and multiple carbonyl resonances (165–180 ppm) is consistent with molecular motifs containing heteroaromatic cores and trione-like functionalities. While the NMR data do not allow an unambiguous identification of HPPT species, the observed spectral features are compatible with their partial formation within the CD system.

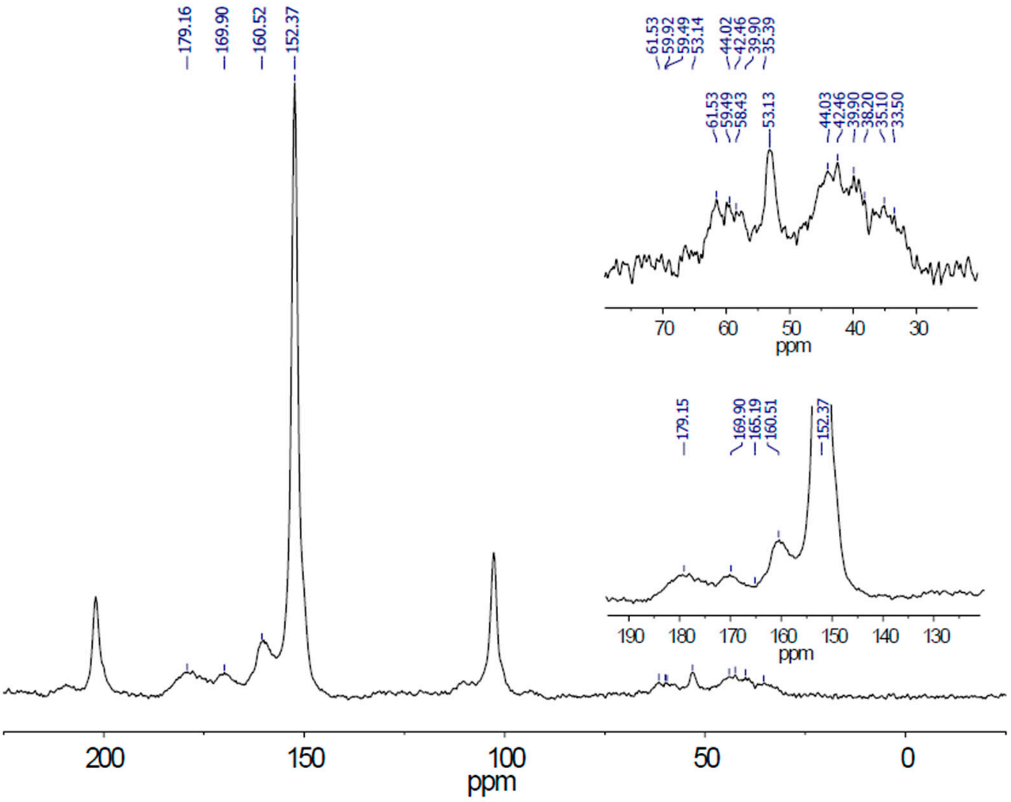

Figure S1. ^{13}C NMR spectrum of CDs synthesized with a U:CA molar ratio of 50:1.
